# High-performance vertical field-effect organic photovoltaics

**DOI:** 10.1038/s41467-023-37174-9

**Published:** 2023-03-22

**Authors:** Xiaomin Wu, Changsong Gao, Qizhen Chen, Yujie Yan, Guocheng Zhang, Tailiang Guo, Huipeng Chen

**Affiliations:** 1 Institute of Optoelectronic Display, National & Local United Engineering Lab of Flat Panel Display Technology, Fuzhou University, Fuzhou, 350002 China; 2 Fujian Science & Technology Innovation Laboratory for Optoelectronic Information of China, Fuzhou, 350100 China; 3 Fujian Provincial Key Laboratory of Quantum Manipulation and New Energy Materials, College of Physics and Energy, Fujian Normal University, Fuzhou, 350007 China; 4 Research Center for Microelectronics Technology, Fujian University of Technology, Fuzhou, 350108 China

**Keywords:** Devices for energy harvesting, Photonic devices

## Abstract

Limited by the inherent energy loss (E_loss_) in carrier transport process, the device efficiency of organic solar cells shows inferior to traditional inorganic photovoltaic devices. Generally, molecular design, morphology optimization and interfacial engineering are usually required to alleviate E_loss_. Here, vertical field-effect organic photovoltaic (VFEOPV) by integrating an bulk-heterojunction (BHJ) organic photovoltaic (OPV) with vertical field effect transistor (VFET) is invented, in which VFET generates a large, uneven, internal electric field, eliminating the requirement for driving force to dissociate excitons and prevents non-radiative recombination in OPV. In this way, the performance of solar cell can be well controlled by the gate voltage of VFET and the E_loss_ of VFEOPVs based on J71: ITIC system is dramatically reduced below 0.2 eV, significantly improving power conversion efficiency (PCE) from 10% to 18% under gate voltage of 0.9 V, which only causes negligible additional power consumption (~10^−4^mJ/cm^2^). Besides, the device also exhibits multi-functionality including transistor and phototransistors with excellent photodector performance. This work provides a new and general strategy to improve the OPV performance which is compatible with present optimization methods, and can be applied to improve PCE of other types of solar cells such as Perovskite and inorganic solar cells.

## Abstract (web summary)

The device efficiency of organic solar cells is usually limited by the inherent energy loss during carrier transport. Here, authors integrate bulk heterojunction organic photovoltaic with vertical field effect transistor, leading to reduced energy loss below 0.2 eV as controlled by the gate voltage.

## Introduction

In the past few years, bulk heterojunction organic photovoltaics (OPV) have achieved dramatically progress and power conversion efficiency (PCE) of single-junction OPV has reached 18.2%^1–6^. However, PCE of organic photovoltaics is still much lower than theoretical value^7,8^. Meanwhile, for most of the reported OPV systems which exhibit PCE > 10%, the synthesis process of materials is extremely complicated and the stability of these materials is far beyond application^9^. Therefore, the general improvement of OPV performance, including both high PCE ones and moderate ones with low cost and high stability, is crucial for the commercialization of OPV.

The PCE of a solar cell is proportional to open-circuit voltage (*V*_OC_), short-circuit current (*J*_SC_) and fill faction (FF). For OPV, *V*_OC_ = (*E*_g_–*E*_loss_)/*e*, where *E*_g_ is the lowest optical bandgap of the donor or acceptor component and *E*_loss_ is photo energy loss. Hence, how to reduce energy loss is the key to improve the *V*_oc_ of solar cell. In single-junction P-N solar cell, the limit Shockley–Queisser PCE is 33%, assuming that no driving force is required for charge separation and there is non-radiative recombination during charge transport, which would lead to *E*_loss_ = 0 eV. However, in OPV, diving force is usually required and non-radiative recombination is inevitable^4,10–12^, resulting in relative large E_loss_. For instance, in low bandgap conjugated system, if efficient charge transport can be realized, the theoretical minimum *E*_loss_ can be 0.6 eV, while E_loss_ of most low bandgap system usually lays in 0.8–0.9 eV^8,9^. Meanwhile, *J*_SC_ and FF are also significantly dependent on energy loss during charge transport^13^. Therefore, reducing the energy loss during charge transport must be able to improve *V*_OC_, *J*_SC_ and FF for the further improvement of PCE. Hence, tremendous efforts have been made to tune the charge transport and recombination process to reduce energy loss, including material design, morphology optimization, and interfacial engineering.

Unfortunately, it is noteworthy that the peak EQE is still considerably below 80% and *E*_loss_ is still larger than 0.8 eV in most of single-junction OPVs due to serious monomolecular/bimolecular charge recombination loss^7–9,14^. Therefore, an alternative strategy to reduce energy/charge recombination loss which is also compatible with present optimization methods is urgent required to further improve PCE of OPVs. Optoelectronic integration which is essential in modern electronic and photonic devices may be an alternative way to solve above issue^15–22^. Transistors founded today’s integrated circuits, information technology, and computer science, which is the core of modern electronic and optoelectronic devices. For instance, in field-effect transistors (FET), the channel current can be precisely controlled by the gate voltage. Therefore, integration of FET with other optoelectronic devices would enhance tunability of the optoelectronic properties of the target devices. Moreover, in science aspect, fundamental understanding of the working mechanism about optoelectronic devices based on filed-effect is very meaning.

Actually, filed-effect has been utilized to control organic light emitting diode (OLED), thermoelectric devices, and optical waveguides^23–35^. For instance, organic light emitting transistor (OLET) integrates organic FET and OLED^18,36^, which utilizes filed-effect to tune the process of charge transport and recombination in OLED to control the lighting. As the working mechanism of OPV and OLED are referential, the success of FET in OLED must lead to the precise control of charge transport and recombination process in OPV using FET. Unfortunately, there is no report about integration of OPV and FET to tune the OPV performance.

Hence, in this work, vertical field-effect transistors (VFET) and OPV are integrated into vertical field-effect organic photovoltaic (VFEOPV). With the effect of tunable gate field, non-radiative recombination is significantly reduced with effective excitons dissociation, leading to significant improvement of PCE with tiny additional power consumption. This work provides a new perspective and system to improve the efficiency of OPV, as well as illustrating how gate field affects the separation of internal exciton, transport of carrier, and the complex physical processes in organic solar cells, which is also applicable to other types of solar cells.

## Results

### Design and fabrication of a VFEOPV

**Fig. 1:**
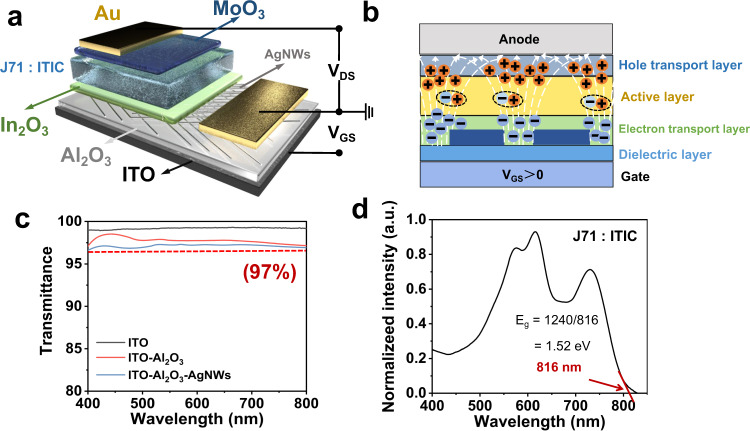
An organic photovoltaic vertical field-effect transistor with high photovoltaic performance. **a**, **b** Device structure of polymer photovoltaic devices with vertical field-effect and a schematic diagram of electric field induced by the gate voltage and the field-assisted charge extraction. In the wavelength range of 400–700 nm, **c** transmittance strength of the device-related reference group. **d** Definition of the Eg of the J71:ITIC film.

The schematic illustration of VFEOPV device structure is presented in Fig. 1a and Supplementary Fig. 1, which integrated an OPV with sandwich structure of Ag nanowires/In_2_O_3_/active layer/MoO_3_/Au and a bottom-gate VFET with vertical current transport. AgNWs are functioned as both cathode of OPV and porous source of VFET. To understand how the VFEOPV work, the working principle diagram is illustrated in Fig. 1b. Gate-electric filed-effect is formed after gate voltage is applied to the insulating layer, finally penetrating into the ETL and active layer. When a positive gate voltage is applied to VFEOPV, a huge density of charges induced by the electric field in the holes of Ag nanowires at the ETL/Ag nanowires interface. A scattered electric filed forms unequal contribution to holes and electrons, obtaining a more balanced charge transport of holes and electrons. The transmittance strength data of the devices were shown in Fig. 1c, which showed a high optical transmittance exceeded 97% in the wavelength range of 400–800 nm. Supplementary Fig. 2 shows scanning electron microscopy (SEM) measurement of Ag nanowires, which exhibits well-distributed condition and excellent connection of metal network structure. According to the observation of atomic force microscope (AFM), J71: ITIC films deposited on Al_2_O_3_ and AgNWs/Al_2_O_3_, as shown in Supplementary Fig. 3a, b, show similar roughness RMS = 2.87 nm and 3.34 nm respectively, which indicates that AgNWs have negligible influence on the morphology of the active layer. Also, the AgNWs height map was tested by AFM, as shown in Supplementary Figs. 4 and 5. To ensure the conductivity of AgNWs to be used as a source, Supplementary Fig. 6 shows the electrical properties of AgNWs, indicating that AgNWs are conductive.

### Electrical performance of the VFEOPV

**Fig. 2:**
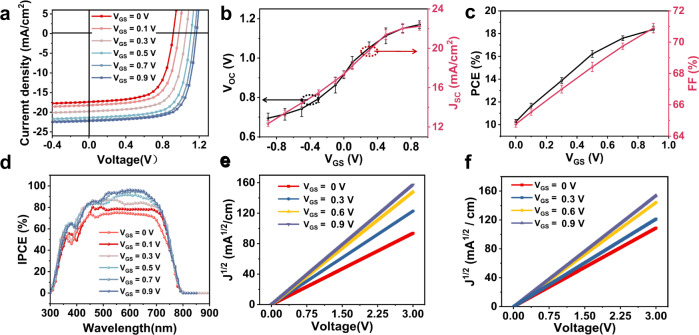
Improvement in photovoltaic performance with vertical field by gate voltage. **a** Current-voltage characteristics of the devices with different *V*_GS_. The dependence of **b** short-circuit current (*J*_SC_) and open circuit voltage (*V*_OC_) and **c** fill factor (FF) and power conversion efficiency (PCE) on gate voltage. **d** IPCE spectra of the corresponding devices with different *V*_GS_, *J*^1/2^–*V* characteristics for **e** hole-only devices, **f** electron-only devices.

The effect of the gate electric field on the performance of the VFEOPV devices with J71:ITIC as the active layer is further investigated and shown in Fig. 2. Figure 2a, Supplementary Figs. 7–10 shows the current-voltage (*I*–*V*) characteristics as a function of gate voltage (*V*_GS_). The results show that the covered area of *I*–*V* curves increases when external *V*_GS_ increases from 0 V to 0.9 V. The extracted performance parameter versus V_GS_ is shown in Fig. 2b, c. *V*_OC_ increases gradually with the increase of positive *V*_GS_ and finally reaches saturation when providing a +0.9 V gate voltage. As the same way, when giving a gradually increased negative *V*_GS_, *V*_oc_ becomes smaller and finally tends to saturation. Similar with *V*_OC_, J_SC_ also increases to saturation along with an increase of FF as gate voltage increases. As a result, PCE shows an increased tendency and gradually to saturation with the increase of external *V*_GS_. A similar trend of the incident photon to converted current efficiency (IPCE) is observed as function of *V*_GS_ at the wavelength from 300 nm to 850 nm, as shown in Fig. 2d, which shows that IPCE increases from 70% to 95% as V_GS_ increases from 0 V to 0.9 V. To examine the effect of electric filed on the transport of hole and electron, the hole mobility and electron mobility are obtained by hole-only and electron- only devices in SCLC measurements, as shown in Fig. 2e, f. The results show that the mobility of both hole and electron increases with the increase of gate voltage, where the electron mobility increases from 2.4 × 10^−8^ m^2^ v^−1^ s^−1^ to 6.9 × 10^−8^ m^2^ v^−1^ s^−1^ while the hole mobility enhances from 3.3 × 10^−8^ m^2^ v^−1^ s^−1^ to 6.6 × 10^−8^ m^2^ v^−1^ s^−1^, which also indicates that the mobility of hole and electron is becoming more balanced.

**Fig. 3:**
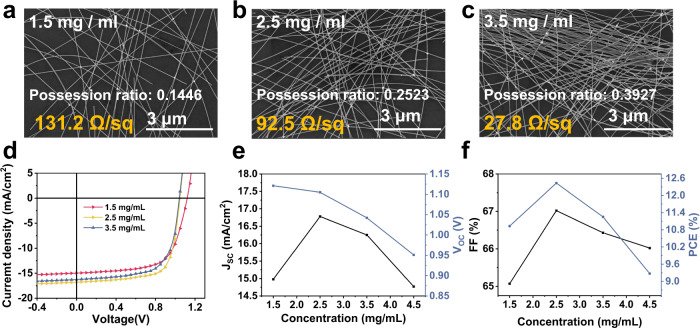
SEM image of AgNWs with different concentration. **a** 1.5 mg/mL, **b** 2.5 mg/mL, **c** 3.5 mg/mL. **d** Current-voltage characteristics of the devices with different concentrations of AgNWs at *V*_GS_ = 0.1 V. **e** short-circuit current (*J*_SC_) and open circuit voltage (*V*_OC_) and **f** fill factor (FF) and power conversion efficiency (PCE) as a function of different AgNWs concentration at *V*_GS_ = 0.1 V.

Since the operation of VFEOPV devices is strongly associated with the penetrable gate electric field through AgNWs cathode, the pore size of AgNWs layer would be a key parameter to affect the electric field intensity and carrier accumulation within the porous region. Hence, the effect of pore size of AgNWs layer on the device performance is investigated. The pore size is measured by the possession ratio of AgNWs. Figure 3a–c shows the SEM images of the cathode AgNWs with different possession ratios at different concentrations. The possession ratio of AgNWs increases from 0.1446 to 0.3927 with the increase in concentration, and the sheet resistance decreases from 131.2 Ω/sq to 27.8 Ω/sq. Figure 3d demonstrates the *I*–*V* characteristics with different concentrations of AgNWs. The results show that *V*_OC_ decreases with an increase in the concentration of AgNWs. Whereas, *J*_SC_ and FF increase firstly and then deteriorate with a peak at 2.5 mg/mL of AgNWs, as shown in Fig. 3e–f. Ultimately, a peak PCE of 12.5% at 2.5 mg/mL of AgNWs with 0.1 V *V*_GS_ is demonstrated. To investigate the influence of cathode area to device performance, the *I*–*V* characteristic was carried out by varying the AgNWs concentration of VFEOPV when *V*_GS_ = 0 V, as shown in Supplementary Figs. 11, 12 and Supplementary Tables 1, 2. The results show that *J*_SC_ increases synchronously with the increasing concentration of AgNWs, while *V*_OC_ and FF barely changed. The results demonstrate that the concentration of AgNWs not only change the cathode area of OPV, but also influence the penetrated electric field and subsequent carrier transport process.

### Working mechanism of the VFEOPV

**Fig. 4:**
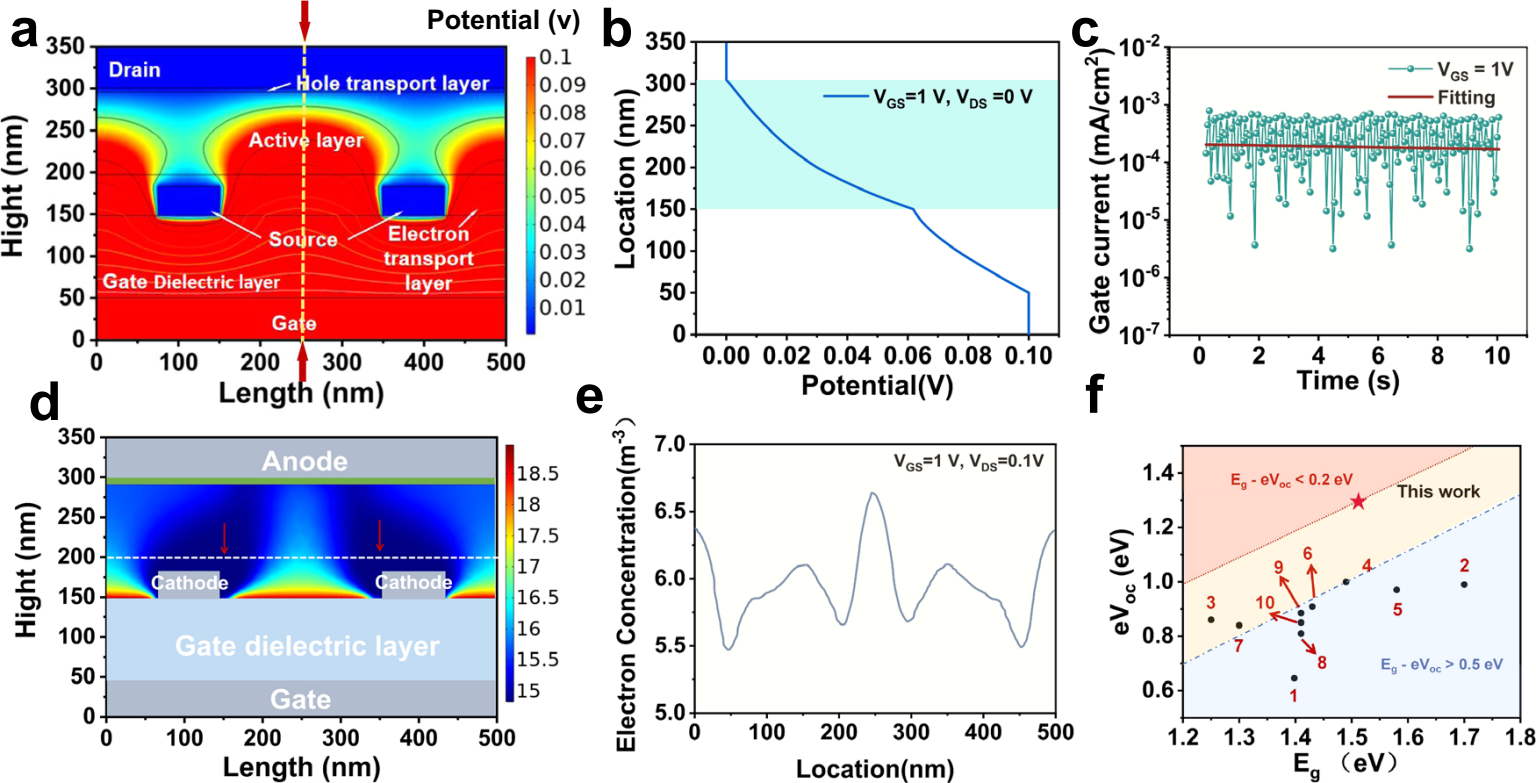
Mechanism analysis of the organic photovoltaic vertical field-effect transistors. **a**, **b** Theoretical simulation of electric potential distribution at *V*_GS_ = 1 V. **c** Gate leakage current of the VFEOPVs. **d**, **e** Theoretical simulation of current density distribution at *V*_GS_ = 1 V. **f** Plots of eV_OC_ against *E*_g_ for organic solar cells with low *E*_loss_ or high *V*oc. The blue dashed line is the *E*_loss_ of 0.5 eV, and the red solid line is the *E*_loss_ = 0.2 eV. The PCEs are shown in parentheses. The number of points corresponds to the structures and device parameters listed in Table S3 in the Supplementary Information.

To further investigate the effect partial screening effects of AgNWs network on the OPV performance, we perform the theoretical simulations by a finite-element calculation using the COMSOL Multiphysics. (Fig. 4a, b and Supplementary Fig. 13 and Supplementary Table 3). In the simulation, AgNWs are grounded, while the gate electrode is applied different voltages from 0.1 V to 1 V. Figure 4a presents the electric potential distribution of conduction channels of VFEOPVs, where the color gradient represents the gate field potential at *V*_GS_ = 1 V. It is apparently seen that the gate field efficiently penetrates into the entire active layer through the empty spaces between the AgNWs. Moreover, the intensity of gate field is no longer homogeneous distributed in channel direction. Figure 4d, e show the electric potential change from anode to gate of VFEOPVs. Another critical issue should be concerned is the additional power consumption induced by gate control, which depends on a to gate leak current (*I*_gl_) in a large extent. As shown in Fig. 4c, the I_gl_ is about the order of magnitude of 10^−4 ^mA/cm^2^, far less than drain source current (*I*_DS_), which is ~10 mA/cm^2^. As a result, the power consumption (*V*_GS_ × *I*_gl_ × *t*) derived from the external electric filed could be negligible compared with that of two-terminal OPV. Moreover, AgNWs covered with In_2_O_3_ could reduce the surface roughness and suppress the joule heat in the junction of AgNWs simultaneously. As shown in Fig. 1d, a low *E*_g_ of 1.52 eV is determined from the onset of the optical absorption in the solid state, which is a commonly accepted and widely used method to determine optical gaps of conjugated polymers. Plots of eV_oc_ as function of *E*_g_ for organic solar cells with low *E*_loss_ or high *V*_oc_ are shown in Fig. 4f. The blue dashed line represents the *E*_loss_ of 0.5 eV, and the red solid line represents the *E*_loss_ = 0.2 eV. The number of the points corresponds to the structures and device parameters listed in Supplementary Table 4 in the Supplementary Information, which further demonstrates that our proposed system is able to obtain an ultra-low *E*_loss._

Moreover, to further demonstrate the applicability of this strategy, other experimental groups of photovoltaic devices based on different types of bulk heterojunction systems coupled with VFET are also explored, as shown in Supplementary Figs. 14, 15 and Supplementary Table 5. As a result, different types of systems exhibit different degrees of PCE when irradiated, which shows that VFETs can couple other types of photovoltaic systems. And the PCE of photovoltaic cells can be further regulated by the gate voltage, which indicates that the field-effect of thin-film transistors can also modulate the energy efficiency of other types of photovoltaic cells. Therefore, this result confirms the high practicality of our strategy to help improve the performance of other types of photovoltaic devices.

Based on the above results, the mechanism of VFEOPV is further illustrated. Supplementary Fig. 16 illustrates the energy-band relationships of the materials in the device. As show in the Supplementary Fig. 17a, photogenerated excitons dissociate into free electrons and holes under built-in electric field. When *V*_GS_ > 0 V, the photogenerated electrons move toward the cathode and accumulate, which lower the Schottky barrier of electrons from the semiconductor to the cathode and promoted the collection of photogenerated charges. As a result, the probability of non-radiative compounding during charge transport is reduced and the open-circuit voltage of the device is enhanced. The increase of *V*_OC_ can be understood by the energy loss (*E*_loss_) mechanisms in OPV. Firstly, the electric filed induced by gate voltage prompts that excitons can be efficiently dissociated with a small driving force, reducing the overall voltage loss. Secondly, the electric filed contributes to the dissociation of charge-transfer (CT) state excitons, thus decreasing the non-radiative recombination, and then reducing *E*_loss_. Thus, the *V*_OC_ can be tuned continuously by controlling the electric field induced by gate voltage. When *V*_GS_ < 0 V, as shown in Supplementary Fig. 17b, the Schottky barrier for electrons entering the cathode from the semiconductor increased, which hindered the collection of photogenerated charges. Therefore, the open-circuit voltage decreased for negative gate voltage compared to positive gate voltage.

The electric field induced by the gate voltage increases the J_SC_ as well. This phenomenon could be ascribed to the controlled dissociation and transport of charge-transfer excitons by field-effect, reducing non-radiative recombination. Hence, IPCE continuously increases with the controlled gate voltage. As well, free-carrier generation is enhanced by increasing dissociation, and the induced electric field is also expected to increase the photogenerated-carrier collection by extending the charge bimolecular-recombination lifetime and increasing the drift length of carriers. Simultaneously, FF is also promoting on account of the unequal contribution to holes and electrons by the scattered electric filed forms, which contributes to the resultant more balanced charge transport of holes and electrons. On the other way, FF expresses the competition of the charge collection and carriers recombination in devices. A reduce of the recombination of carriers promotes the FF sequentially. Furthermore, the improvement of OPV by the external electric filed could be explained by the electrical doping effect. The larger built-in potential induced by *V*_GS_ enables a more efficient collection of carriers, which results in a larger FF. An inevitable factor limiting the performance of OPV is *E*_loss_, which is related to the exciton dissociation and the radiative and non-radiative recombination. As previous articles have reported, there could be a trade-off between minimizing the *E*_loss_ and the photogeneration. A low driving force could results in a high *V*_OC_ with slight *E*_loss_. However, a low driving force causes inefficient charge generation and low quantum efficiency, limiting the photo-current. There is no solution to solve this issue until now, while in this work, IPCE still increases with high photo-current when V_OC_ improves simultaneously even with a very low driving force.

### Applying to high-performance photodetector functionality

**Fig. 5:**
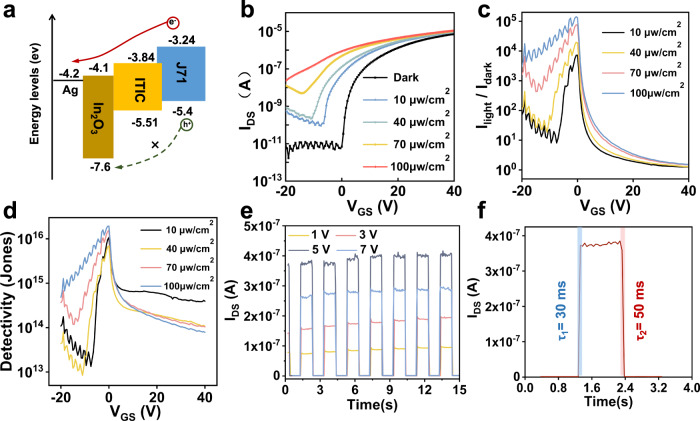
Hybrid BHJ polymers—In_2_O_3_ phototransistors. **a** Energy diagram of the charge-separated electron and hole transport between the J71:ITIC and In_2_O_3_ interface. **b** Photoresponse of the J71:ITIC/In_2_O_3_ devices under the light intensity in the range of 10−100 μW cm^−2^. **c, d** Responsivity and detectivity of J71:ITIC/In_2_O_3_ devices at different light intensity. **e** Amplified photocurrent response of J71:ITIC/In_2_O_3_ phototransistors with V_DS_ set to 1, 3, 5, or 7 V, at a pulse frequency of 0.5 Hz. **f** The real-time temporal photocurrent response during on-off illumination switching.

Furthermore, according to the result, the non-radiative recombination in the channel is effectively reduced, it is implied that the device has the potential to be applied with high-performance phototransistors. Therefore, the phototransistor performance of the device is systematically investigated, which demonstrate ultra-high photosensitive photodetector characteristics under 400–780 nm irradiation. The transfer curve and output curve of the VFEOPV devices were shown in Supplementary Fig. 9, which revealed a threshold voltage *V*_TH_ of 1.8 V. Figure 5a demonstrates the energy band of the system and suggests that the electron transport in the channel can be enhanced while the holes are blocked by In_2_O_3_, which effectively improves the separation of photogenerated charges and obtains excellent photosensitivity. In order to confirm the photosensitivity characteristics of the J_71_:ITIC/ In_2_O_3_ phototransistors, we examined the electrical variations in the devices under the illumination of different power density (10–100 μW cm^−2^), which are shown in Fig. 5b. Evidently, a huge negative shift of the threshold voltage is obtained relative to the dark condition, which is desirable for phototransistor to obtain high photo gain. The evaluations of the phototransistors are performed by using responsivity (*R*), detectivity (D*), and response speed. The responsivity and detectivity are given by Eq. (1) and Eq. (2). Therefore, we calculated the detectivity using the Noise Equivalent Power (NEP),

1 $${{{{{\rm{R}}}}}}=\frac{{I}_{{ph}}}{{P}_{{inc}}A}$$

2 $${D}^{*}=\frac{\sqrt{A\triangle f}}{{NEP}}$$

3 $${{{{{\rm{NEP}}}}}}=\frac{{i}_{n}}{R}$$

where A is the effective area of the detector, $\triangle f$ is the electrical bandwidth, and ${i}_{n}$ is the noise current. As a result, the device exhibits a responsivity over 10^5^, as shown in Fig. 5c, and a detectivity of 1.99 × 10^16^ Jones exceeding most phototransistors (Supplementary Table 6), as presented in the Fig. 5d. According to the excellent result, the mechanism is discussed as following. Firstly, given the vertical structure, the extremely short transport distance would greatly promote the separation of photogenerated exciton at the donor-acceptor interface. During this process, the photogenerated electrons are affected by the positive *V*_GS_ to accumulate toward the insulating layer/In_2_O_3_ interface, while the gate electric field that across the AgNWs lowers the Schottky barrier, which promotes the injection of electrons to increase the *I*_DS_^37–39^. On the other hand, due to the output current direction in the vertical transistor parallel to the vertical channel direction and the role of In_2_O_3_ in blocking the holes while enhancing electron transport, the high-intensity electric field strength in the channel under the applied *V*_DS_ allows the holes to be rapidly extracted by the drain, which greatly reduces the non-radiative recombination of the photogenerated holes with the injected electrons and thus further increases the photocurrent^40–42^. Moreover, Fig. 5e shows the optical switching characteristics of the device at different *V*_DS_ and demonstrates fast saturation and recovery with rise and fall times of 30 ms and 50 ms, respectively, as presented in Fig. 5f, which further demonstrates that the combination of vertical structure and energy-band matching greatly suppresses recombination at the J71: ITIC/In_2_O_3_ interface and enhances electron transport.

Finally, the superiority of VFEOPV is summarized: (i) Ultra-low E_**loss**_. Trade-off between minimizing the *E*_loss_ and the photogeneration (main challenge in OPV) is solved by VFEOPV. By integrating the OPV with vertical field-effect transistor, the generated a large, uneven internal electric field eliminates the requirement for driving the separated exciton and prevents non-radiative recombination. As a result, the device achieves an ultra-low *E*_loss_ = 0.2 eV, which is better than reported work. (ii) Negligible additional power consumption. Negligible additional power consumption (~10^−4^ mJ/cm^2^), which is far less than that of two-terminal OPV is caused to significantly improve PCE from 10% to 18%. The generality of this method is also verified (Supplementary Fig. 15). (iii) Multi-functionality. VFEOPV exhibits multi-functionality including transistor and phototransistors with excellent photodector performance (detectivity is over 1.99 × 10^16^ Jones and responsivity is over 10^5^), which exceeds most phototransistors. (iv) High degree of compatibility. This work provides a new and general strategy to improve the OPV performance which is compatible with present optimization methods such as materials design and morphology optimization, and can be applied to improve PCE of other types of solar cells such as Perovskite and inorganic solar cells.

## Discussion

In summary, a high-performance VFEOPV was first reported by integrating organic photovoltaic with vertical field-effect transistors. Different from the traditional methods such material design and morphology optimization, we propose to use gate voltage field-effect to regulate the performance of solar cells, and systematically elucidated the effect of field-effect modulation on physical mechanisms such as carrier transport and recombination in solar cells. Due to the nanometer-scale channel length, the uneven electric field in the VFET can greatly promote the separation and transport of photogenerated carriers, which effectively reduces the non-radiative complex and increases the EQE and improves the *J*_SC_. Besides, the additional electric field can effectively reduce the internal driving force required for carrier separation, which reduced the *E*_loss_ during transmission and thus improves *V*_oc_. As a result, the device demonstrated an ultra-low *E*_loss_ of below 0.2 eV and PCE is significantly improved from 10% to 18% under gate voltage of 0.9 V, which only causes negligible additional power consumption compared to the consumption of two-terminated-OPV. Moreover, VFEOPV exhibits multi-functionality including transistor and phototransistors with excellent photodector performance, which exceeds most phototransistors. Therefore, this work provided an innovative and compatible strategy for designing and upgrading high-performance OPVs and is applicable to other types of optoelectronic devices including solar cells, which have great potential in the development of high-performance photovoltaic devices and smart energy.

## Methods

### Materials and fabrication

Precursor ink for indium oxide (In_2_O_3_) was prepared by dissolving In(NO_3_) _3_ · xH _2_O in anhydrous 2-methoxyethanol (Sigma-Aldrich 99.8%) in 0.2 M concentration (6.8 wt% of In(NO_3_)_3_ · xH_2_O). Then 200 μL of acetylacetone (Acac, Sigma, 99%) and 70 μL of ammonium hydroxide solution (NH3·H_2_O, 28% NH_3_ in H_2_O) were added as additives. The solution was stirred at 75 °C for >12 h and filtered prior to using a 0.45-μm pore size glass fiber filter. J71 and ITIC were purchased from 1-Materials. As shown in Supplementary Fig. 18, a layer of Al_2_O_3_ after 100 nm was deposited on the ITO electrode substrate as an insulating layer by the ALD technique. Then, the silver nanowire solution as the cathode was spin-coated on the Al_2_O_3_ insulating layer at 2000 rpm to construct the silver nanowire network electrode. It was annealed at 100 °C for 60 s to remove the residual solvent. Then, 50 nm of gold was vaporized onto the silver nanowires by thermal vapor deposition through the mask version as a contact electrode for the silver electrode to easily connect to the probe during testing.

The indium oxide film with 40 nm as ETL and channel layer was fabricated by inkjet-printing after O_2_ Plasma treatment with 5 min. After inkjet-printing of an indium precursor solution, the devices were dried at 150 °C to remove off the residual solvents and then annealed at 240 °C in air. Solution of the active layer was prepared by dissolving J71 (10 mg/mL) and ITIC (10 mg/mL) in chlorobenzene. The active layers ~100 nm were fabricated by inkjet-printing at the jetting voltage of 60 V in a substrate temperature of 80 °C, and then thermally annealed subsequently at 150 °C for 10 min in nitrogen glove box. Finally, MoO_3_ and top Au electrode were evaporated under high vacuum ~5.0 × 10^−5^ Pa with a shadow mask. The experimental sections for the other experimental groups in Supplementary Fig. 14 were recorded in Supplementary Note 1.

### Equipment and characterization

The current density-voltage (*I*–*V*) characteristics of the devices were measured under AM 1.5 G (100 mW/cm^2^) using Sun 2000, Abet Technologies. The hole and electron mobility of the blend films were measured using the space charge limited current (SCLC) method with device structures of AgNWs/PEDOT:PSS/active layer /MoO_3_/Au (hole-only device) and AgNWs /In_2_O_3_/PFN/active layer/PFN/Au (electron-only device). A depth profiler (BRUKER, DektakXT) was been applied to measure the film thickness, and SEM (Nova NanoSEM 230) measurement was performed to obtain the distribution of Ag nanowires (cathode) on Al_2_O_3_. Atomic force microscopy (AFM) (Bruker NanoScope V) was employed to characterize the surface morphology of active layers.

### Reporting summary

Further information on research design is available in the Nature Portfolio Reporting Summary linked to this article.

## Supplementary information

Supplementary Information

Solar Cells Reporting Summary

## Data Availability

The data that support the plots within this paper and other findings of this study are available from the corresponding authors upon request.

## Code availability

The code that supports the theoretical plots within this paper is available from the corresponding author upon request.
