# Supplementary material for: High-performance vertical field-effect organic photovoltaics
Source: Nat Commun. 2023 Mar 22;14:1579. doi: 10.1038/s41467-023-37174-9 (PMC10033512; doi:10.1038/s41467-023-37174-9)
Supplement: Supplementary file 1 — Supplementary Information [file 41467_2023_37174_MOESM1_ESM.pdf]

Supplementary information

High-performance vertical Field-Effect Organic Photovoltaics

Xiaomin Wu[1,2,3,†], *Changsong Gao*[1,2,†], *Qizhen Chen*[1,2], *Yujie Yan*[1,2], *Guocheng Zhang*[1,4], *Tailiang Guo*[1,2], *Huipeng Chen*[1,2,*]

[1]Institute of Optoelectronic Display, National & Local United Engineering Lab of Flat Panel Display Technology, Fuzhou University, Fuzhou 350002, China

[2]Fujian Science & Technology Innovation Laboratory for Optoelectronic Information of China, Fuzhou 350100, China

[3]Fujian Provincial Key Laboratory of Quantum Manipulation and New Energy Materials, College of Physics and Energy, Fujian Normal University, Fuzhou, 350007, China

[4]Research Center for Microelectronics Technology, Fujian University of Technology, Fuzhou 350108, China

[†] These authors contributed equally to this work.

*Corresponding author

Email: hpchen@fzu.edu.cn

Supplementary Figure 1： Organic photovoltaic devices combined with field-effect transistor devices.

Supplementary Figure 2. The SEM image of AgNWs deposited on Al_2O_3/ITO.

Supplementary Figure 3. The effect of AgNW on the morphology of Al_2O_3.

Supplementary Figure 4. Height map of AgNWs tested by atomic force microscopy.

Supplementary Figure 5. Measuring the height of AgNWs.

Supplementary Figure 6. Electrical properties of the AgNW used in the experiments.

Supplementary Figure 7. Transfer curve of FET based on In_2O_3 annealed under 240℃.

Supplementary Figure 8. Mobility of In_2O_3 as a function of annealing temparature.

Supplementary Figure 9. Transistor characteristics of VFEOPV.

Supplementary Figure 10. Current-voltage characteristics of the devices with different $V_{GS}<0$ V.

Supplementary Figure 11. Current-voltage characteristics of the devices with different concentration of AgNWs at $V_{GS} = 0$ V.

Supplementary Figure 12. Battery performance based on different concentrations of AgNWs.

Supplementary Figure 13. Theoretical simulation of electric potential distribution at $V_{GS} = 0.1$ V.

Supplementary Figure 14. Current-voltage characteristics of devices with different blend active layers devices.

Supplementary Figure 15. PCE histogram for different blends under $V_{GS} = 0$ V and $V_{GS} = 2$ V.

Supplementary Figure 16. The schematic diagram of the material energy band structure in the device.

Supplementary Figure 17. The energy band relationship after being influenced by the gate electric field.

Supplementary Figure 18. The technological preparation process of VFEOPVs.

Supplementary Note 1. Experimental details of other experimental groups based on different types of bulk heterojunction systems.

Supplementary Table 1. Device performance of OPV with V_{GS} = 0.1 V under simulated AM1.5G illumination.

Supplementary Table 2. Device performance of OPV with V_{GS} = 0 V under simulated AM1.5G illumination.

Supplementary Table 3. COMSOL related simulation parameters.

Supplementary Table 4. Performance of different types of organic solar cells in other work.

Supplementary Table 5. Performance of devices with different blend active layers.

Supplementary Table 6. Key performance parameters of reported phototransistors.

High-performance vertical Field-Effect Organic Photovoltaics

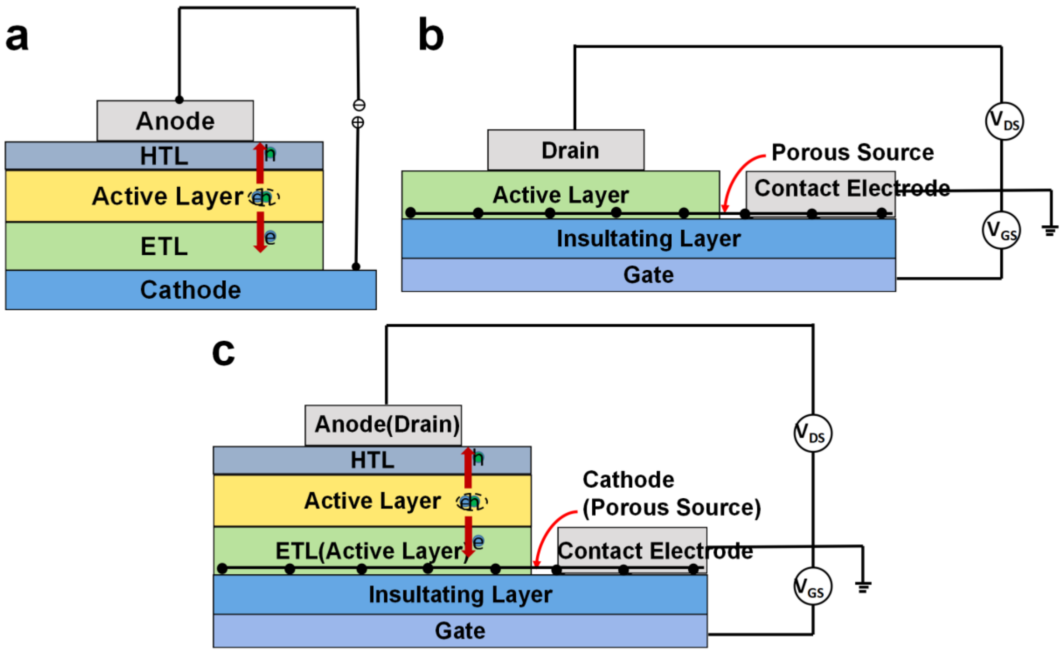

Supplementary Figure 1. Organic photovoltaic devices combined with field-effect transistor devices. Device structure of **a** organic photovoltaics (OPV), **b** vertical field effect transistors (VFET), **c** an vertical field effect organic photovoltaics (VFEOPVs).

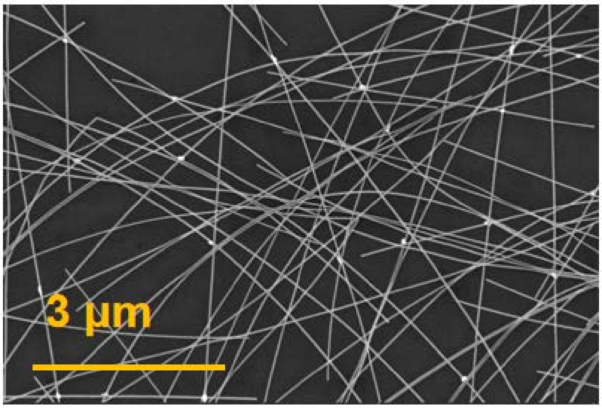

Supplementary Figure 2. The SEM image of AgNWs deposited on Al_2O_3/ITO.

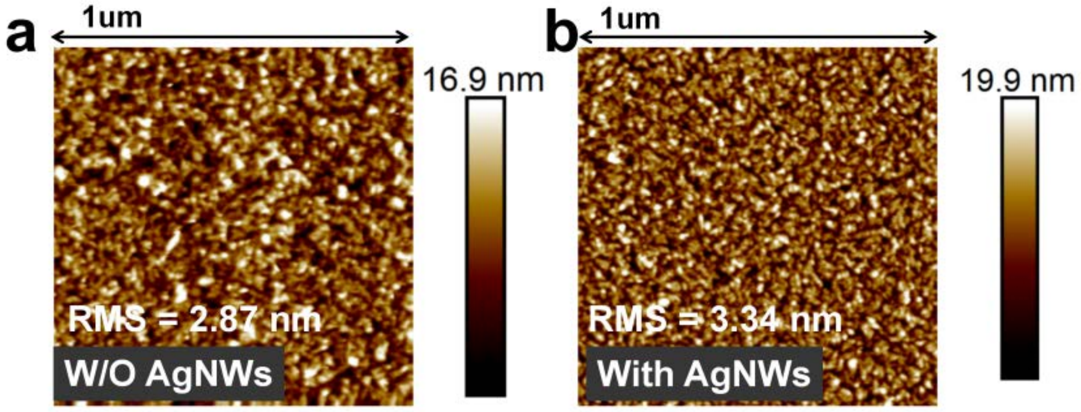

Supplementary Figure 3. The effect of AgNW on the morphology of Al_2O_3. AFM image of J71:ITIC deposited on **a** Al_2O_3 and **b** AgNWs/Al_2O_3.

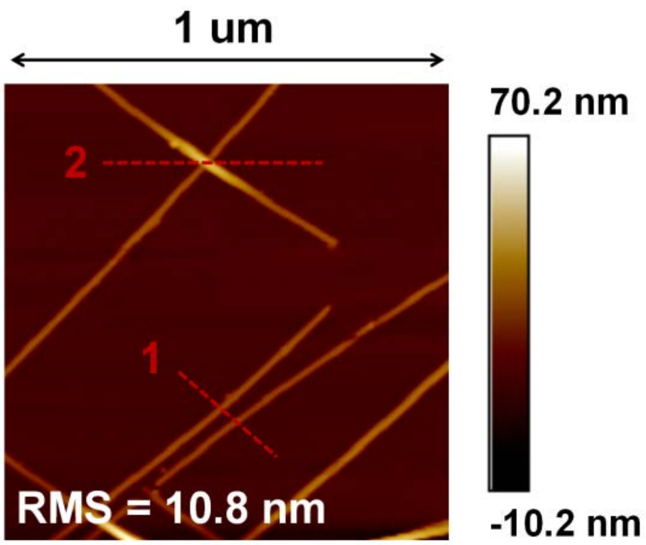

Supplementary Figure 4. Height map of AgNWs tested by atomic force microscopy.

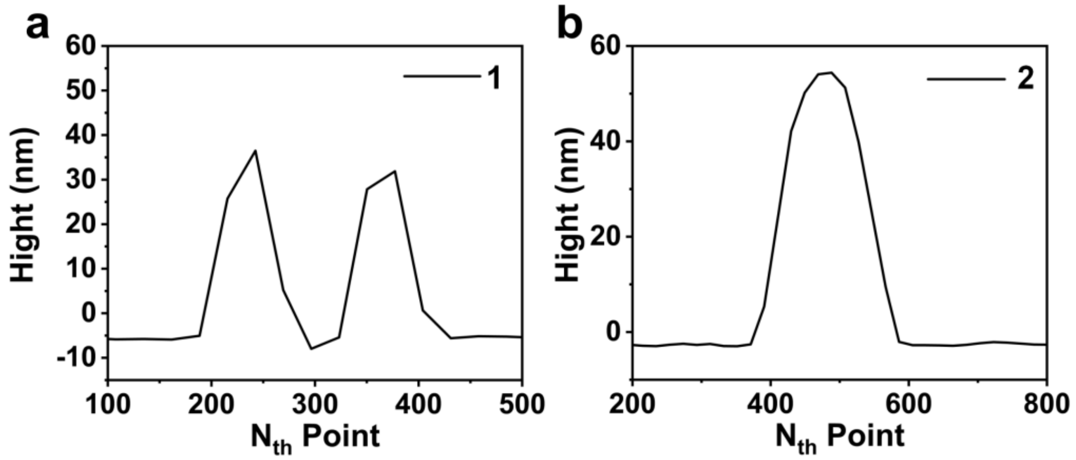

Supplementary Figure 5. Measuring the height of AgNWs. a Height of single AgNW and **b** overlapping areas.

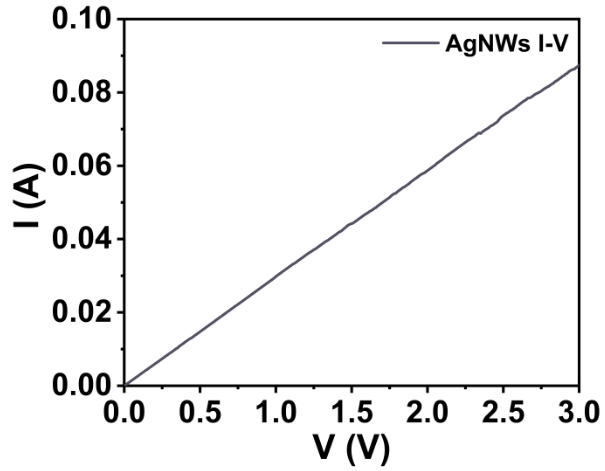

Supplementary Figure 6. Electrical properties of the AgNW used in the experiments.

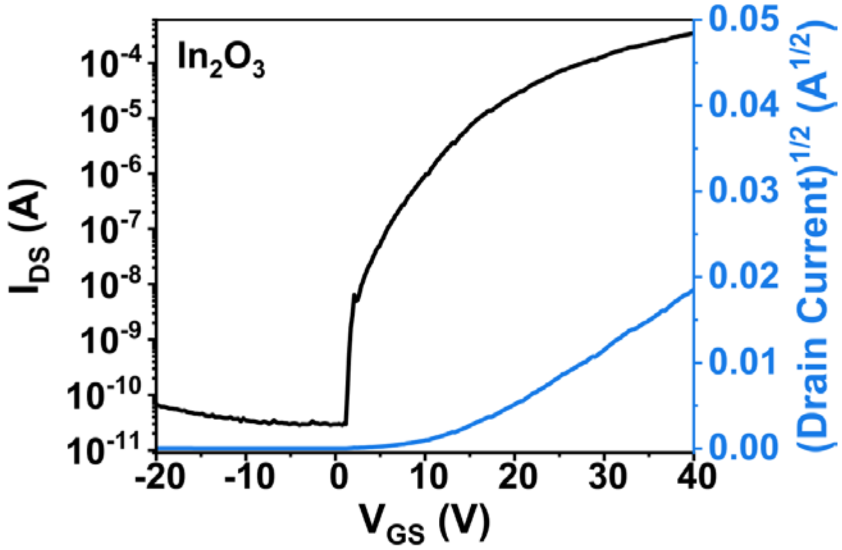

Supplementary Figure 7. Transfer curve of FET based on In_2O_3 annealed under 240°

C.

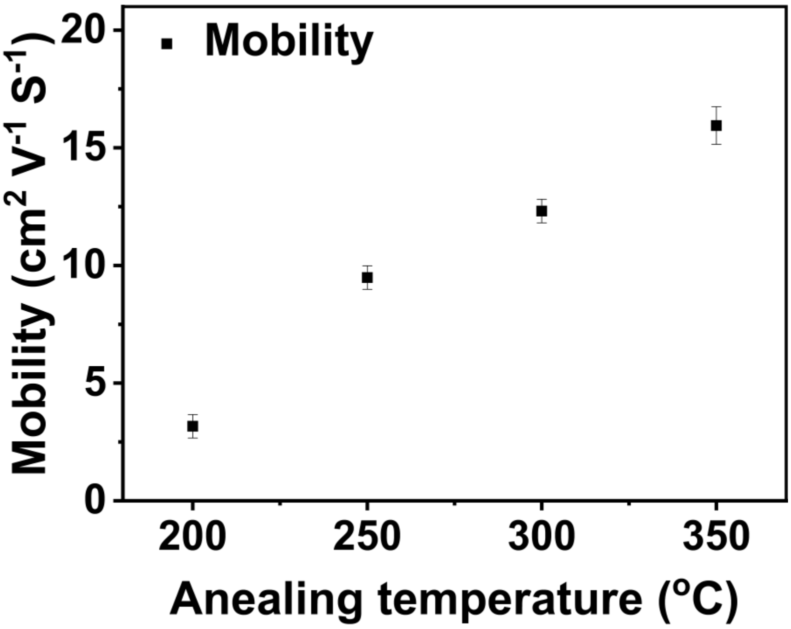

Supplementary Figure 8. Mobility of In_2O_3 as a function of annealing temparature.

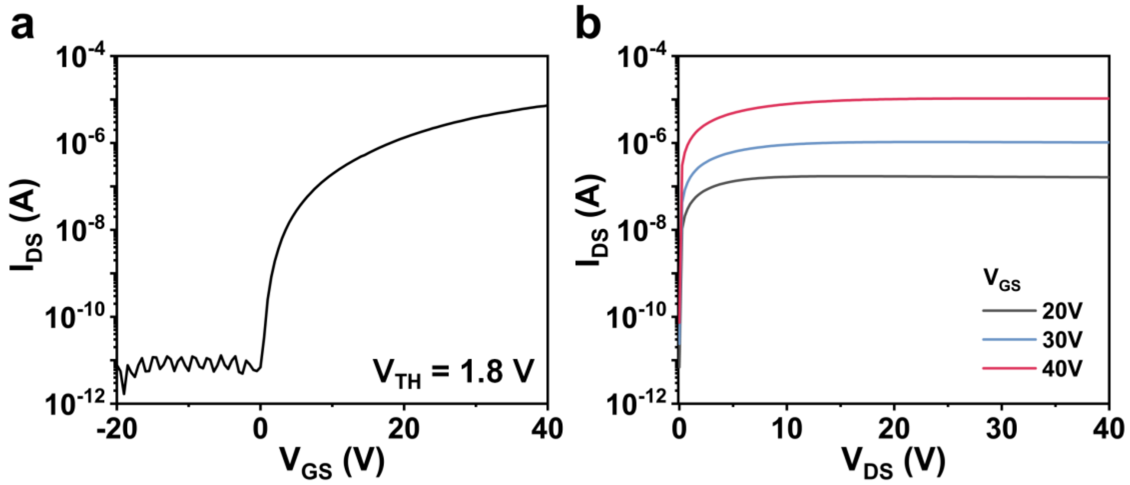

Supplementary Figure 9. Transistor characteristics of VFEOPV. a Transfer curve of the device. **b** Output curve of the device.

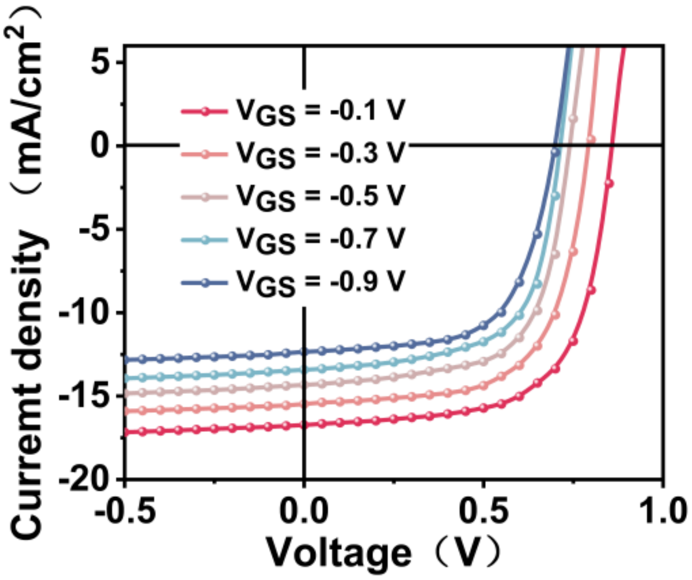

Supplementary Figure 10. Current–voltage characteristics of the devices with different V_{GS}<0 V.

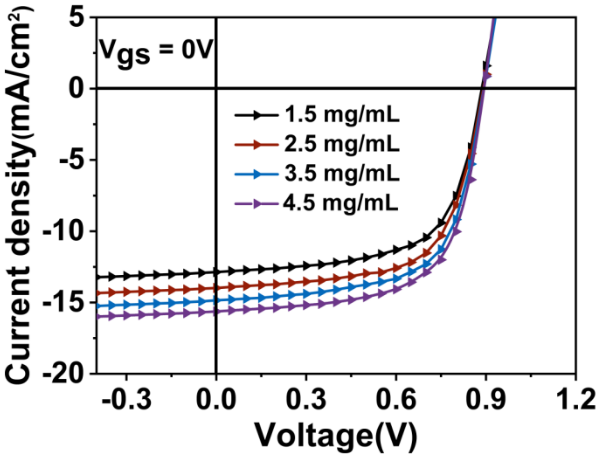

Supplementary Figure 11. Current–voltage characteristics of the devices with different concentration of AgNWs at V_{GS} = 0 V.

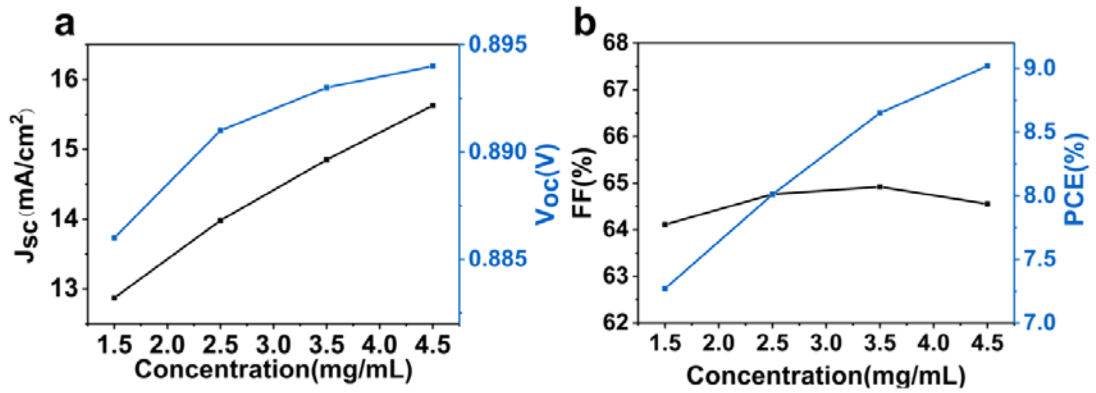

Supplementary Figure 12. Battery performance based on different concentrations of AgNWs. a short circuit current (J_{sc}) and open circuit voltage (V_{oc}), **b** fill factor (FF) and power conversion efficiency (PCE) as a function of different AgNWs concentration at V_{GS} = 0 V.

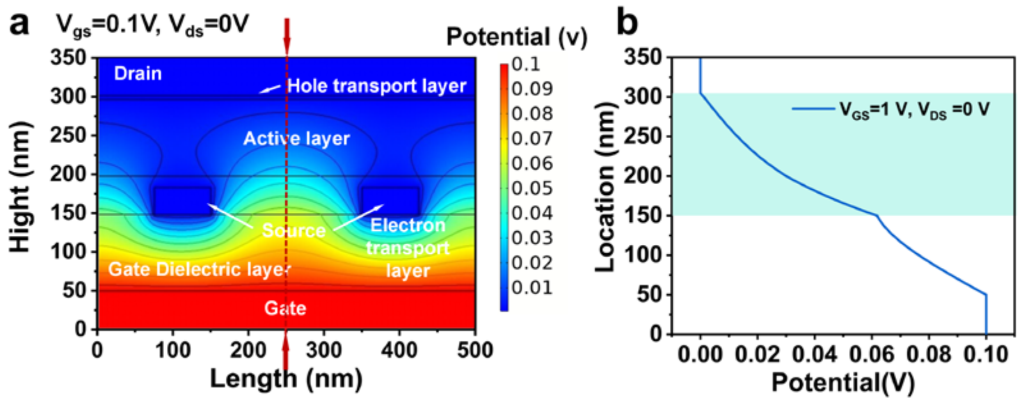

Supplementary Figure 13. Theoretical simulation of electric potential distribution at V_{GS} = 0.1 V. a The potential distribution inside the device at V_{GS} = 0.1 V, V_{DS} = 0 V. **b** The potential distribution at the red line of the device at V_{GS} = 0.1 V, V_{DS} = 0 V.

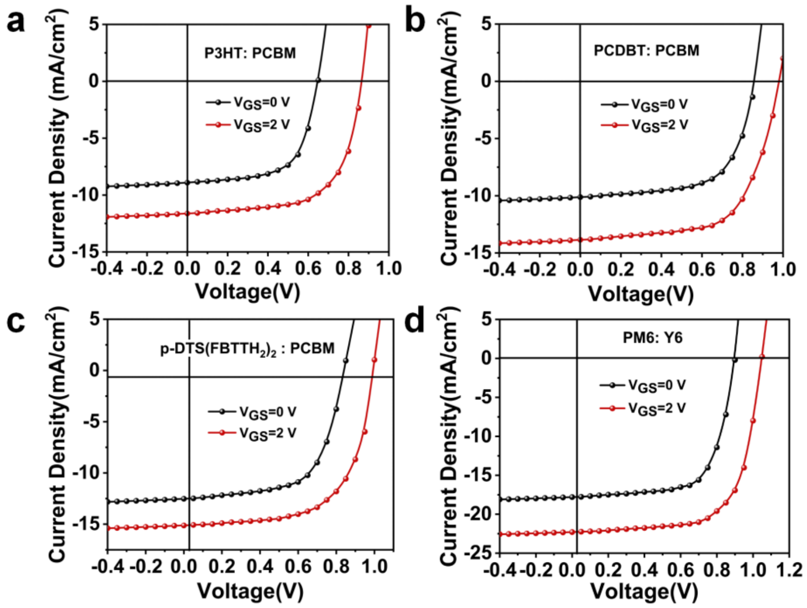

Supplementray Figure 14. Current–voltage characteristics of devices with different blend active layers devices. a P3HT : PCBM. **b** PCDBT : PCBM. **c** p-DTS(FBTTH$_2$)$_2$: PCBM. **d** PM6 : Y6.

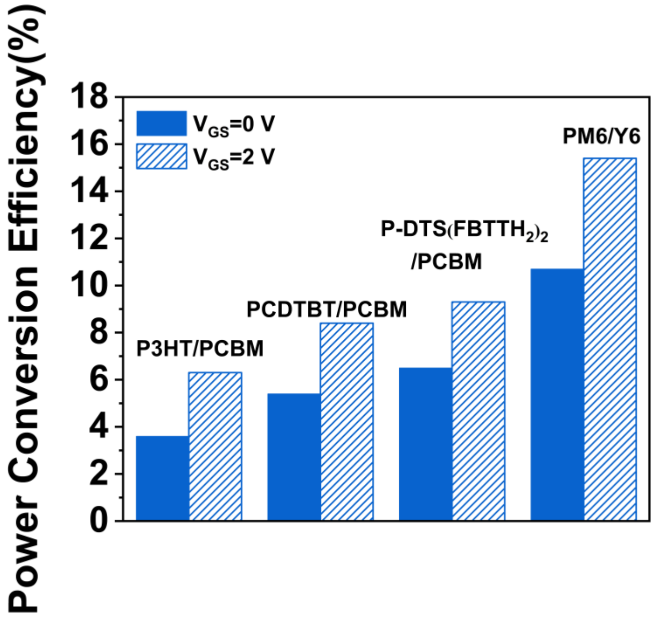

Supplementary Figure 15. PCE histogram for different blends under V_{GS} = 0 V and V_{GS} = 2 V.

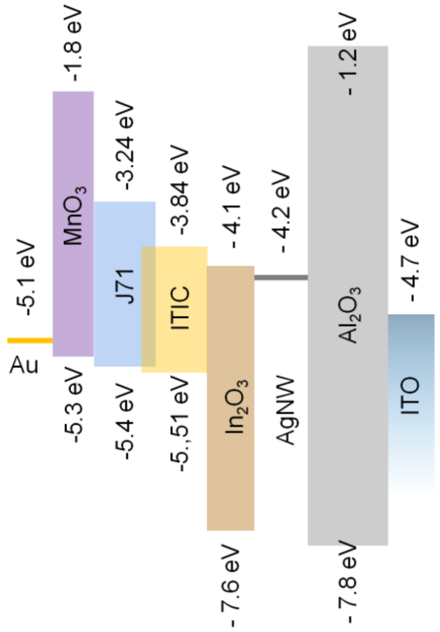

Supplementary Figure 16. The schematic diagram of the material energy band structure in the device.

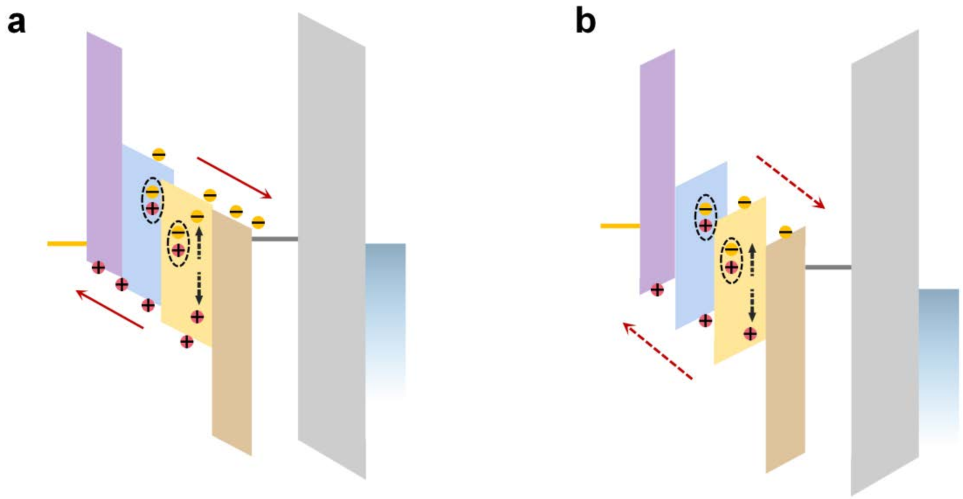

Supplementary Figure 17. The energy band relationship after being influenced by the gate electric field. Energy band relationship **a** when Vgs > 0 V and **b** when Vgs < 0 V.

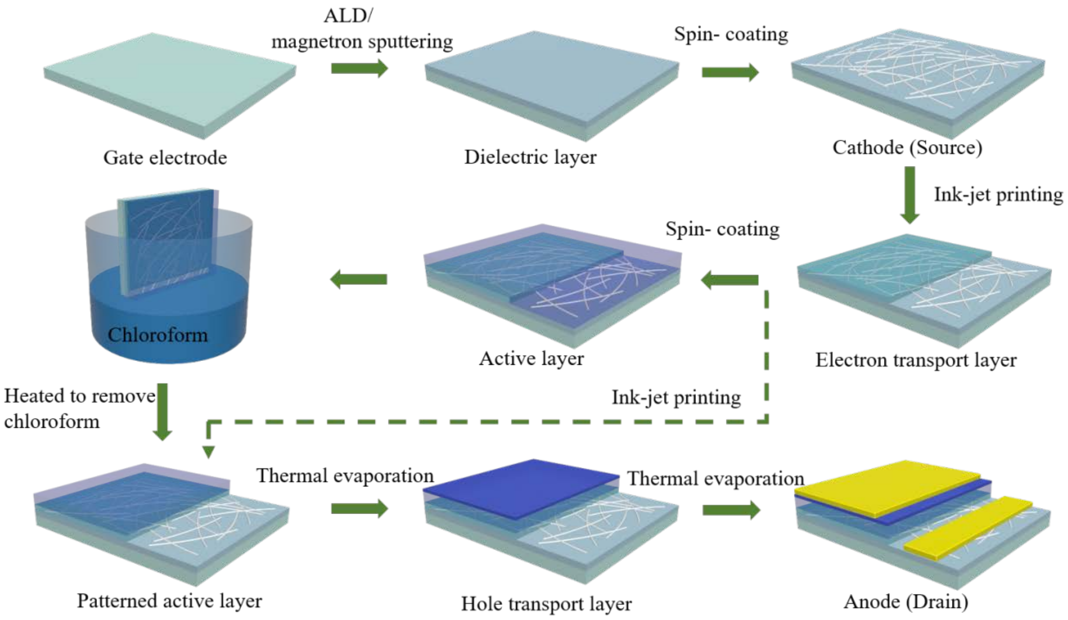

Supplementary Figure 18. The technological preparation process of VFEOPVs.

Supplementary Note 1

Experimental details of other experimental groups based on different types of bulk heterojunction systems.

The [6,6]-phenyl-C61-butyric acid methyl ester(PCBM), poly{[N,N′-bis(2-octyldodecyl)- naphthalene1,4,5,8-bis(dicarboximide)-2,6-diyl]-alt-5,5′-(2,2′-bithiophene)} (N2200), poly[N-9′-heptadecanyl-2,7-carbazole-alt-5,5-(4′,7′-di-2-thienyl-2′,1′,3′-benzothiadiazole)] (PCDTBT) and 7,7′-(4,4-bis(2-ethylhexyl)-4H-silolo[3,2-b:4,5-b′]dithiophene-2,6-diyl)bis(6-fluoro-4(5′-hexyl-[2,2′-bithiophen]-5-yl)benzo[c][1,2,5]thiadiazole) (P-DTS(FBTTH$_2$)$_2$) were purchased from Derthon Optoelectric Materials Science Technology Co. Ltd. and used without further purification. The Y6 and PM6 ere purchased from Sigma-Aldrich. A silver (Ag) nanowire solution (5mg ml^{-1} in isopropanol) was purchased from Suzhou Cold Stone Nano Material Technology Co.

The Ag nanowires had a diameter and length of about 40 ± 5 nm and 30 ± 5 um, respectively, and were diluted to a concentration of 0.5 mg/mL with isopropanol for the formation of the mesh source electrode. A layer of Al_2O_3 after 100 nm was deposited on the ITO electrode substrate as an insulating layer by the ALD technique. Then, the silver nanowire solution as the cathode was spin-coated on the Al_2O_3 insulating layer at 2000 rpm to construct the silver nanowire network electrode. It was annealed at 100℃ for 60 s to remove the residual solvent. Then, 50 nm of gold was vaporized onto the silver nanowires by thermal vapor deposition through the mask version as a contact electrode for the silver electrode to easily connect to the probe during testing.

For the experimental group with P3HT/PCBM blended heterojunction as the active layer. The poly(3,4-ethylenedioxythiophene) polystyrene sulfonate (PEDOT:PSS) layer was spun on a mesh source electrode at 1000 rpm for 60 s,

followed by drying in air at 110 °C for 2 min and then annealing at 130 °C for 8 min under N_2 atmosphere, and removed the redundant film covering the contact electrode with chloroform. Then, the solution of active layer was prepared by dissolving P3HT (20 mg/mL) and PCBM (15 mg/mL) (1:0.8) in chlorobenzene. The active layers were fabricated by inkjet-printing at the jetting voltage of 70 V in a substrate temperature of 80 °C, and then thermal annealed subsequently at 150 °C for 10 min in nitrogen glove box.

For the experimental group with PCDTBT/PCBM blended heterojunction as the active layer. The PEDOT:PSS layer was spun on a mesh source electrode at 1000 rpm for 60 s, followed by drying in air at 110 °C for 2 min and then annealing at 130 °C for 8 min under N_2 atmosphere. Then, the solution of active layer was prepared by dissolving PCBM (15 mg/mL) and PCDTBT (10 mg/mL) (1:0.6) in chlorobenzene. The active layers were fabricated by inkjet-printing at the jetting voltage of 60V in a substrate temperature of 80 °C, and then thermal annealed subsequently at 120 °C for 15 min in nitrogen glove box.

For the experimental group with p-DTS(FBTTH$_2$)$_2$/PCBM blended heterojunction as the active layer. The PEDOT:PSS layer was spun on a mesh source electrode at 1000 rpm for 60 s, followed by drying in air at 110 °C for 2 min and then annealing at 130 °C for 8 min under N_2 atmosphere. Then, the solution of active layer was prepared by dissolving PCBM (15 mg/mL) and p-DTS(FBTTH$_2$)$_2$ (12 mg/mL) (1:0.8) in chlorobenzene. The active layers were fabricated by inkjet-printing at the jetting voltage of 70V in a substrate temperature of 80 °C, and then thermal annealed subsequently at 135 °C for 10 min in nitrogen glove box.

For the experimental group with P3HT/PCBM blended heterojunction. The PEDOT:PSS layer was spun on a mesh source electrode at 1000 rpm for 60 s, followed by drying in air at 110 °C for 2 min and then annealing at 130 °C for 8 min under N_2 atmosphere. Then, the solution of active layer was prepared by dissolving PM6 (10 mg/mL) and Y6 (10 mg/mL) (1:1) in chlorobenzene. The active layers were

fabricated by inkjet-printing at the jetting voltage of 60 V in a substrate temperature of 70 °C, and then thermal annealed subsequently at 150 °C for 12 min in nitrogen glove box. Finally, 50 nm gold was thermally evaporated through a shadow mask as the drain electrode.

The effective channel area (2 mm × 2 mm) was determined by the overlapping area between the Ag nanowires and top gold drain electrode.

Concentration	J_{SC} (mA/cm^2)	V_{OC} (V)	FF (%)	PCE (%)
1.5mg/mL	14.98±0.3	1.121±0.04	65.07±0.4	10.93±0.3
2.5mg/mL	16.78±0.3	1.105±0.04	67.02±0.5	12.43±0.3
3.5mg/mL	16.25±0.4	1.042±0.05	66.43±0.4	11.25±0.3

Supplementary Table 1. Device performance of OPV with V_{GS} = 0.1 V under simulated AM1.5G illumination.

Concentration	J_{SC} (mA/cm^2)	V_{OC} (V)	FF (%)	PCE (%)
1.5mg/mL	12.87±0.3	0.886±0.04	64.11±0.4	7.27±0.3
2.5mg/mL	13.98±0.2	0.891±0.03	64.76±0.3	8.01±0.3
3.5mg/mL	14.85±0.4	0.893±0.05	64.92±0.5	8.65±0.5
4.5mg/mL	15.63±0.4	0.894±0.06	64.55±0.5	9.02±0.5

Supplementary Table 2. Device performance of OPV with V_{GS} = 0 V under simulated AM1.5G illumination.

	X	Y	Relative permittivity
Gate (ITO)	500 nm	50 nm	-
Dielectric layer (Al_2O_3)	500 nm	100 nm	9.3
Source (AgNWs)	75 nm	35 nm	4
In_2O_3 (ETL)	500 nm	50 nm	1.8
Active layer	500 nm	100 nm	2.3
MoO_3 (HTL)	500 nm	5 nm	1.8
Drain (Au)	500 nm	50 nm	-

Supplementary Table 3. COMSOL related simulation parameters.

Number	Active blend	Eg (eV)	eVoc (eV)	Eloss （eV）	PCE(%)	Reference
1	PCBM:PBDBTF:Y6	1.398	0.645	0.553	16.5	1
2	Polymers(P1,P2,P3): PC71BM	1.7	0.99	0.71	10.3	2
3	PBDB-T:ITIC/PEDOT:PSS/$(FASnI_3)_{0.6}(MAPbI_3)_{0.4}$	1.25	0.86	0.39	18.03	3
4	PIDTT-TID :$PC_{71}BM$	1.49	1	0.49	6.7	4
5	PBDS-T:ITIC	1.58	0.97	0.61	11	5
6	PNDT-ST_x:PBDT-$ST_{(1-x)}$:Y6-$T_{1.2}$	1.43	0.909	0.521	16.57	6
7	APDC-TPDA:Y6	1.3	0.84	0.46	16.96	7
8	P2F-EHp:Y6	1.41	0.81	0.6	16.02	8
9	PBDT-T-SF:Y6:ITCT	1.41	0.885	0.525	16.14	9
10	PM_6:Y6:$PC_{61}BM$	1.41	0.85	0.56	16.67	10
11	J71:ITIC	1.52	1.334	0.186	18.46	This work

This work was tested with V_{GS} =1.5 V.

Supplementary Table 4. Performance of different types of organic solar cells in other work.

	Condition	J_{SC} (mA/cm^2)	V_{OC}(V)	FF(%)	PCE(%)
P3HT:PCBM	V_{GS}=0 V	8.9± 0.3	0.65± 0.02	63.7± 0.2	3.6± 0.2
	V_{GS}=2 V	11.6± 0.2	0.86± 0.04	64.1± 0.3	6.3± 0.3
PCDBT:PCBM	V_{GS}=0 V	10.12± 0.3	0.85± 0.03	63.8± 0.3	5.4± 0.2
	V_{GS}=2 V	13.85± 0.2	0.98± 0.04	63.4± 0.4	8.4± 0.3
P-DTS(FBTTH$_2$)$_2$:PCBM	V_{GS}=0 V	12.52± 0.4	0.84± 0.02	63.2± 0.2	6.5± 0.2
	V_{GS}=2 V	15.12± 0.2	0.99± 0.02	63.3± 0.4	9.3± 0.2
PM6:Y6	V_{GS}=0 V	17.8± 0.3	0.96± 0.03	68.2± 0.2	10.7± 0.3
	V_{GS}=2 V	22.2± 0.3	1.05± 0.04	67.5± 0.3	15.4± 0.3

Supplementary Table 5. Performance of devices with different blend active layers.

Device	Materials	R(A/W)	D^*(Jones)	Intensity(μW/cm^2)	Voltage(V)	Ref.
Organic Materials	PCDTBT:PC71BM/DNTT	480	N/A	40	15	12
	C8-BTBT	393	N/A	20	70	13
	pentacene/$PC_{61}BM$	0.33	N/A	530	80	14
	pentacene/PTCDI-C_8	1×10^{-2}	1.26×10^{9}	107	60	15
	PDVT-8:PCBM	750	4.45×10^{15}	100	40	16
2D Materials	Multilayer MoS_2	4×10^{4}	2.9×10^{13}	210	40	17
	p-n MoS2	7.0×10^{4}	$\approx 3.5\times10^{14}$	0.001	40	18
	WS_2/Graphene	2.2×10^{5}	3.5×10^{13}	34	2.5	19
	C8-BTBT/Graphene	1.56×10^{4}	N/A	10	10	20
Perovskite	Graphene:Perovskite	3.3×10^{10}	2.71×10^{13}	25	15	21
	SWNTs:Perovskite	6.0×10^{4}	2.46×10^{14}	900	20	22
	Organic-inorganic Perovskite	1.7×10^{4}	2.0×10^{14}	1000	20	23
	Perovskite Nanowire/Graphene	2.6×10^{6}	N/A	0.05	60	24
Organic/Inorganic Materials	J71:ITIC/In_2O_3	1.43×10^{5}	1.93×10^{16}	10	7	This work

Supplementary Table 6. Key performance parameters of reported phototransistors.

Supplementary References

1. Yu, R., *et al.* Improved Charge Transport and Reduced Nonradiative Energy Loss Enable Over 16% Efficiency in Ternary Polymer Solar Cells. *Advanced Materials* **31**, 1902302 (2019).
2. Zhu, D., *et al.* Single-junction fullerene solar cells with 10% efficiency and high open-circuit voltage approaching 1 V. *Nano Energy* **40**, 495-503 (2017).
3. Xu, G., *et al.* Integrating Ultrathin Bulk-Heterojunction Organic Semiconductor Intermediary for High-Performance Low-Bandgap Perovskite Solar Cells with Low Energy Loss. *Advanced Functional Materials* **28**, 1804427 (2018).
4. Wang, C., *et al.* Low Band Gap Polymer Solar Cells With Minimal Voltage Losses. *Advanced Energy Materials* **6**, 1600148 (2016).
5. Huang, B., *et al.* Alkylsilyl Functionalized Copolymer Donor for Annealing-Free High Performance Solar Cells with over 11% Efficiency: Crystallinity Induced Small Driving Force. *Advanced Functional Materials* **28**, 1800606 (2018).
6. Xu, X., *et al.* Subtle Polymer Donor and Molecular Acceptor Design Enable Efficient Polymer Solar Cells with a Very Small Energy Loss. *Advanced Functional Materials* **30**, 1907570 (2020).
7. Du, X., *et al.* Delayed Fluorescence Emitter Enables Near 17% Efficiency Ternary Organic Solar Cells with Enhanced Storage Stability and Reduced Recombination Energy Loss. *Advanced Functional Materials* **30**, 1909837 (2020).
8. Fan, B., *et al.* Achieving over 16% efficiency for single-junction organic solar cells. *Science China Chemistry* **62**, 746-752 (2019).
9. Deng, W., *et al.* EMSCs Build an All-in-One Niche via Cell-Cell Lipid Raft Assembly for Promoted Neuronal but Suppressed Astroglial Differentiation of Neural Stem Cells. *Adv Mater* **31**, e1806861 (2019).
10. Chang, Y., *et al.* A 16.4% efficiency organic photovoltaic cell enabled using two donor polymers with their side-chains oriented differently by a ternary strategy. *Journal of Materials Chemistry A* **8**, 3676-3685 (2020).
11. Yan, T., *et al.* 16.67% Rigid and 14.06% Flexible Organic Solar Cells Enabled by

Ternary Heterojunction Strategy. *Advanced Materials* **31**, 1902210 (2019).

12. Pierre, A., *et al.* Charge-integrating organic heterojunction phototransistors for wide-dynamic-range image sensors. *Nature Photon* **11**, 193–199 (2017).

13. Huang, J. *et al.* Printable and Flexible Phototransistors Based on Blend of Organic Semiconductor and Biopolymer. *Advanced Functional Materials* **27**, 1604163−1604171 (2017).

14. Labram, J. *et al.* Low-voltage ambipolar phototransistors based on a pentacene/PC61BM heterostructure and a self-assembled nanodielectric. *Organic Electronics* **11**, 1250−1254 (2010).

15. Kim, J. S.*et al.* Schottky-Barrier-Controllable Graphene Electrode to Boost Rectification in Organic Vertical P-N Junction Photodiodes. *Advanced Functional Materials* **27**, 1704475−1704482 (2017).

16. Jian, Z., *et al.* High Performance Flexible Organic Phototransistors with Ultrashort Channel Length. *ACS Photonics* **5**, 3712-3722, (2018).

17. Sunwoo, H., *et al.* Performance enhancement of multilayer MoS2 phototransistors via photoresist encapsulation. *Current Applied Physics* **41**, 14-17 (2022)

18. Huo, N., *et al.* Ultrasensitive all-2D MoS_2 phototransistors enabled by an out-of-plane MoS2 PN homojunction. *Nat Communications* **8**, 572 (2017).

19. Lee, W. *et al.* Photoinduced Tuning of Schottky Barrier Height in Graphene/MoS2 Heterojunction for Ultrahigh Performance Short Channel Phototransistor. *ACS Nano* **14**, 7574-7580 (2020).

20. Liu, X., *et al.* Epitaxial Ultrathin Organic Crystals on Graphene for High-Efficiency Phototransistors. *Advanced Materials* **28**, 5200-5205 (2016).

21. Yu, S. *et al.* Stable Graphene-Two-Dimensional Multiphase Perovskite Heterostructure Phototransistors with High Gain. *Nano Letters* **17**, 7330-7338 (2017).

22. Liu, Z. *et al.* Photoresponsive Transistors Based on Lead-Free Perovskite and Carbon Nanotubes. *Advanced Functional Materials* **30**, 1906335 (2020).

23. Chen, Y., *et al.* High-Performance Inorganic Perovskite Quantum Dot–Organic

Semiconductor Hybrid Phototransistors. *Advanced Materials* **29**, 1704062 (2017).
24. Spina, M., *et al.* Photodetectors: Microengineered CH3NH3PbI3 Nanowire/Graphene Phototransistor for Low-Intensity Light Detection at Room Temperature. *Small* **11**, 4823-4823 (2015).
