# Supplementary material for: High-performance vertical field-effect organic photovoltaics
Source: Nat Commun. 2023 Mar 22;14:1579. doi: 10.1038/s41467-023-37174-9 (PMC10033512; doi:10.1038/s41467-023-37174-9)
Supplement: Supplementary file 2 — Solar Cells Reporting Summary [file 41467_2023_37174_MOESM2_ESM.pdf]

nature research

Corresponding author(s): Huipeng Chen

Solar Cells Reporting Summary

Nature Research wishes to improve the reproducibility of the work that we publish. This form is intended for publication with all accepted papers reporting the characterization of photovoltaic devices and provides structure for consistency and transparency in reporting. Some list items might not apply to an individual manuscript, but all fields must be completed for clarity.

For further information on Nature Research policies, including our data availability policy, see Authors & Referees.

Experimental design

Please check: are the following details reported in the manuscript?

1. Dimensions

Item	Yes	No	Response
Area of the tested solar cells	☒	☐	The area of the device is 2mm * 2mm.
Method used to determine the device area	☒	☐	Area is defined by the optical microscope.

2. Current-voltage characterization

Item	Yes	No	Response
Current density-voltage (J-V) plots in both forward and backward direction	☐	☒	Hysteresis is not observed in this organic solar cell, so only focus on the forward test
Voltage scan conditions<br>*For instance: scan direction, speed, dwell times*	☒	☐	We used a dwell time of 30 ms for each voltage and a step voltage of 0.02 V (forward)
Test environment<br>*For instance: characterization temperature, in air or in glove box*	☒	☐	Test at room temperature 25 celsius degree in air environment
Protocol for preconditioning of the device before its characterization	☐	☒	No preconditioning protocol was applied before characterization
Stability of the J-V characteristic<br>*Verified with time evolution of the maximum power point or with the photocurrent at maximum power point; see ref. 7 for details.*	☐	☒	Not analysed, the stability is not a critical issue for the claims and conclusion of the study.

3. Hysteresis or any other unusual behaviour

Item	Yes	No	Response
Description of the unusual behaviour observed during the characterization	☐	☒	No unusual behavior
Related experimental data	☐	☒	N.A.

4. Efficiency

Item	Yes	No	Response
External quantum efficiency (EQE) or incident photons to current efficiency (IPCE)	☒	☐	In Figure 2 of the main manuscript.
A comparison between the integrated response under the standard reference spectrum and the response measure under the simulator	☐	☒	This is not involved in this study
For tandem solar cells, the bias illumination and bias voltage used for each subcell	☐	☒	The series connection between batteries is not involved in this work.

5. Calibration

Item	Yes	No	Response
Light source and reference cell or sensor used for the characterization	☒	☐	The current density-voltage (J-V) characteristics of the devices were measured under AM 1.5G (100 mW/cm2) using Sun 2000, Abet Technologies. The light intensity was calibrated with a single-crystal Si-based solar cell.
Confirmation that the reference cell was calibrated and certified	☒	☐	The reference cell was calibrated and certified

Calculation of spectral mismatch between the reference cell and the devices under test

- [ ] Yes
- [x] No

The spectral mismatch factor was not considered

6. Mask/aperture

Size of the mask/aperture used during testing

- [ ] Yes
- [x] No

we didn't use masks during testing in the lab

Variation of the measured short-circuit current density with the mask/aperture area

- [ ] Yes
- [x] No

The influence of device area is not related to this study, but we have kept the device area always being the same.

7. Performance certification

Identity of the independent certification laboratory that confirmed the photovoltaic performance

- [ ] Yes
- [x] No

This is not involved in this study

A copy of any certificate(s)
Provide in Supplementary Information

- [ ] Yes
- [x] No

This is not involved in this study

8. Statistics

Number of solar cells tested

- [x] Yes
- [ ] No

We eested 16 cells for each device with standard error shown in Table S1.

Statistical analysis of the device performance

- [x] Yes
- [ ] No

Statistical analysis of the device performance is shown is Figure 3, Table S1 and S2.

9. Long-term stability analysis

Type of analysis, bias conditions and environmental conditions
For instance: illumination type, temperature, atmosphere humidity, encapsulation method, preconditioning temperature

- [ ] Yes
- [x] No

Not analyzed, because long-term stability is not a critical issue for the claims and conclusion of this study.
